# Supplementary material for: Netcombin: An algorithm for constructing optimal phylogenetic network from rooted triplets
Source: PLoS One. 2020 Sep 18;15(9):e0227842. doi: 10.1371/journal.pone.0227842 (PMC7500971; doi:10.1371/journal.pone.0227842)
Supplement: S4 File — (ZIP) [file pone.0227842.s004.zip › SequenceData-Triplet/sequenceData-10-20-30-0-Triplet/length 400, numseq 40, rate 10^-7/2 seq.pdf]

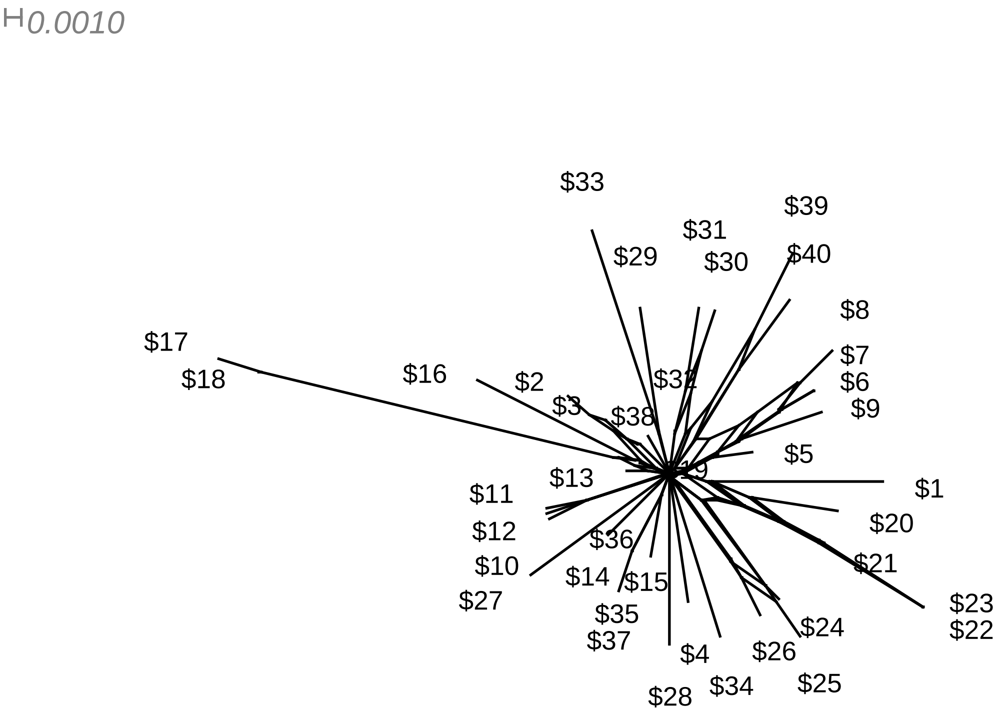

0.0010
$33
$39
$31
$29
$40
$30
$8
$17
$7
$16
$18
$6
$2
$32
$9
$3
$38
$5
$13
$1
$11
$20
$12
$36
$21
$10
$14
$15
$27
$23
$35
$22
$24
$37
$4
$26
$34
$25
$28
